# Turn up the Vitamin D Receptor—Not the Calcium! Photoswitchable Vitamin D Agonists for Psoriasis

**DOI:** 10.1021/acscentsci.5c02264

**Published:** 2025-12-16

**Authors:** Cyril Goudet, Olalla Vázquez

**Affiliations:** † IGF, Univ Montpellier, CNRS, Inserm, Montpellier, France; ‡ Chemistry Department, Marburg University, Hans-Meerwein-Straße 4, 35043 Marburg, Germany; § Centre for Synthetic Microbiology (SYNMIKRO), Karl-von-Frisch-Straße 14, 35043 Marburg, Germany

## Abstract

The azobenzene-containing agonist of the vitamin D receptor, PhotoVDRM, enables spatiotemporal psoriasis treatment in mice; its *cisoid* form induces potent anti-inflammation without hypercalcemia.

## Graphical abstract

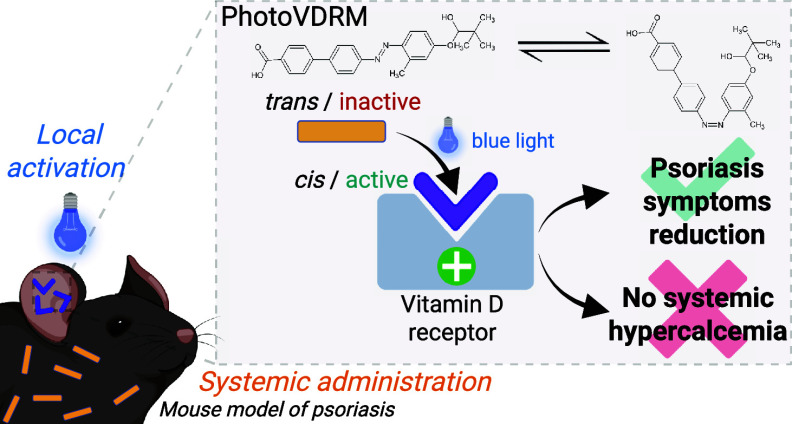

Photopharmacology is an emerging interdisciplinary field at the interface of pharmacology and photochemistry that takes advantage of molecular transducers capable of undergoing reversible isomerization, i.e., photoswitches. By incorporating photoswitches into bioactive molecules, light can exert spatiotemporal control over compound activity. In this context, azobenzene-based switches are currently the most commonly employed, due to their synthetic accessibility as well as suitable photochemical properties. Importantly, the photoisomerization of azobenzene from the thermodynamically stable *trans* isomer to the metastable *cis* one entails distinct geometry (transition from a planar one to a more compact, globular one; decrease in the end-to-end distance from ∼9 Å to ∼6 Å) and electronics (increase in the dipole moment from ∼0D to ∼3D) that turn azobenzene into highly effective scaffolds for the photocontrol of biological systems.^1,2^ From a therapeutic perspective, an ideal light-operated drug should be inactive in the dark and become active only under irradiation, limiting its action to the illuminated targeted area in order to avoid off-target effects. Unfortunately, conventional azobenzene-based photodrugs often display suboptimal profiles because, typically, the thermally stable trans isomer is the active form (“*trans*-on”).^3^ To address this limitation, we could either use the sign inversion approach using cyclic diazocines or develop *cis*-on molecules with the ability to interact with the receptor only in the *cis* configuration (*cisoid* geometry), resembling the active agonists, whereas the *trans* flat structure is more distant from the active ligand geometries.

In this issue of *ACS Central Science*, Rovira, Krishnan, Llebaria, and their colleagues introduce an innovative strategy to treat psoriasis by developing *cis*-on photoswitchable agonists of the vitamin D receptor (VDR), which enables a light-controlled localized and time-limited treatment of psoriasis in preclinical mouse models without inducing the undesirable side effect of hypercalcemia (Figure 1).^4^

**Figure 1:**
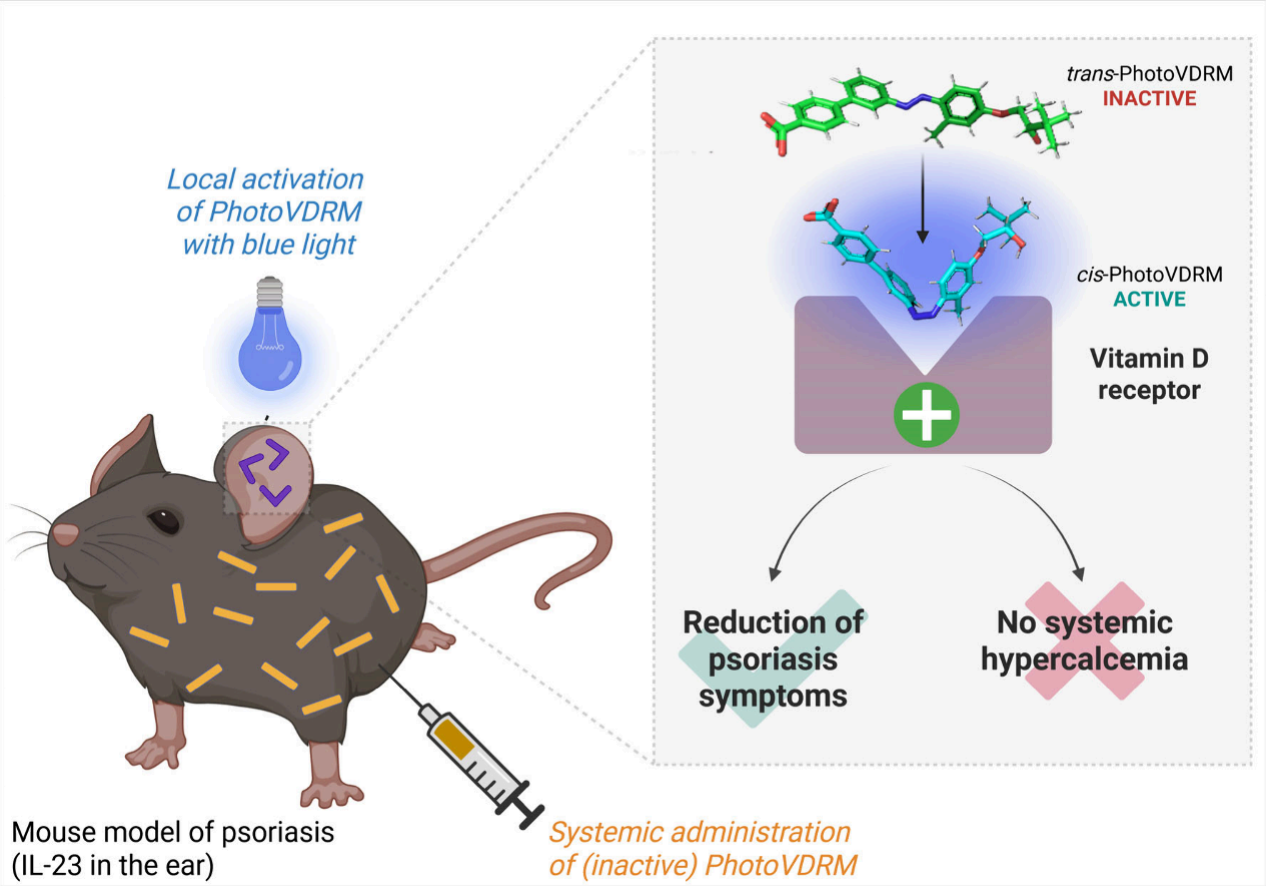
A photoswitchable agonist of the vitamin D receptor to treat psoriasis with high spatiotemporal precision. PhotoVDRM is an azobenzene-containing agonist of the vitamin D receptor, which is inactive in the dark in its *trans* configuration but can be precisely activated by UV and blue light to become a highly potent agonist in its *cis* configuration. It enables a light-controlled localized and time-limited treatment of psoriasis in a preclinical mouse model, without inducing the undesirable side effect of hypercalcemia. Created with Biorender.com.

Conventional azobenzene-based photodrugs often display suboptimal profiles because, typically, the thermally stable *trans* isomer is the active form (“*trans*-on”). To address this limitation, we could either use the sign inversion approach using cyclic diazocines or develop *cis*-on molecules with the ability to interact with the receptor only in the *cis* configuration (*cisoid* geometry), resembling the active agonists, whereas the *trans* flat structure is more distant from the active ligand geometries.

Psoriasis is one of the most common dermatological diseases, affecting approximately 100 million people worldwide.^5^ It is a chronic autoimmune illness characterized by persistent inflammation and hyperproliferation of keratinocytes, leading to the formation of red, scaly patches with potential systemic complications. Beyond physical discomfort, psoriasis can have profound psychological and social repercussions that greatly affect the quality of life of those affected.^6^ Psoriasis is a multifactorial medical condition, influenced by both environmental and genetic factors, although its exact etiology remains unclear. While there is no cure for psoriasis yet, several treatments help in symptomatology relief. On the one hand, severe cases require systemic interventions based on immunosuppressants, biotherapies targeting inflammatory molecules, or oral retinoids. On the other hand, mild to moderate psoriasis is typically treated with topical agents such as corticosteroids, vitamin D derivatives, keratolytics, and phototherapy. The intrinsic accessibility of this skin illness offers a unique advantage for light treatments. Phototherapy, especially narrowband UV-B and bath-psoralen UV-A, is a mainstay of psoriasis treatment.^7^ It acts through anti-inflammatory and antiproliferative effects on keratinocytes, with a good tolerance profile. UV-B triggers the conversion of 7-dehydrocholesterol into vitamin D_3_, which is subsequently hydroxylated into the steroid hormone calcitriol that activates the VDR. This receptor modulates both calcium absorption and disease-relevant immune pathways, reducing inflammation.^8^ Recent advances aim to improve the accessibility and safety of treatments, notably by employing nontoxic blue or red light in dynamic phototherapy, combined with local photosensitizers for targeted applications.

Despite recent advances in developing VDR agonist clinical candidates with reduced calcemic effects, the distinction between therapeutic efficacy and calcium-elevating activity remains inadequate to permit oral administration for the treatment of conditions such as osteoporosis, cancer, leukemia, and psoriasis.

Besides narrowband UV-B phototherapy, photopharmacology has recently demonstrated the therapeutic benefits of targeting receptors involved in psoriasis, like the A_3_ adenosine receptor.^9^ Along these lines, vitamin D and its receptor modulate inflammation, keratinocyte proliferation, and immune regulation, making them major therapeutic targets in psoriasis.^10^ Vitamin D deficiency is common in patients with psoriasis and has been associated with disease severity. By modulating the immune system and limiting the production of pro-inflammatory cytokines, vitamin D reduces skin inflammation. Vitamin D and its analogues (such as calcipotriol and calcitriol) act directly on keratinocytes to inhibit their proliferation and promote their normal maturation, thereby reducing the thickness and plaque extent. These analogues are widely used in topical applications for mild to moderate psoriasis, often combined with corticosteroids to enhance efficacy and reduce side effects. Therefore, activation of VDR has a clear therapeutic benefit in psoriasis, but despite recent advances in developing VDR agonist clinical candidates with reduced calcemic effects, the distinction between therapeutic efficacy and calcium-elevating activity remains inadequate to permit oral administration for the treatment of conditions such as osteoporosis, cancer, leukemia, and psoriasis.

Combining the benefits of phototherapy with targeted activation of VDRs could open up a new therapeutic avenue to tackle psoriasis in the future.

To develop a library of *cis*-on VDR agonists, the team analyzed over 100 VDR–ligand crystal structures, including the nonsecosteroidal diarylmethane family of agonists and calcitriol. In particular, they grafted an azobenzene moiety onto LSN2148936, retaining the *t*-butyl phenoxymethyl carbinol side chain and incorporating a biphenyl group to maintain the critical contacts ideally only in the *cisoid* conformation. Twelve photoswitchable VDR agonists were synthesized via standard procedures. Of note, the authors also included enantiomerically pure compounds using chiral hydroxyanilines. Gratifyingly, all compounds demonstrated the desired photopharmacological profile with better activity in their *cis* configuration at the concentration assayed. The lead compound, PhotoVDRM, exhibited negligible activity across a broad concentration range (1 nM–10 μM) in the absence of irradiation. However, light turned it into the most potent agonist, with a pEC_50_ in the same range as the parental LSN2148936 (pEC50_*cis*‑PhotoVRDM_ 7.23 vs pEC50_LSN2148936_ 7.80). As predicted by molecular modeling, hydrogen–deuterium exchange mass spectrometry (HDX-MS) under light and dark conditions confirmed the interaction of the *cis* isomer with key residues in the ligand-binding domains. Importantly, PhotoVDRM displayed rapid photoisomerization kinetics, yielding high *cis* ratios within seconds even at low light intensities. PhotoVDRM could also be repeatedly switched without loss of efficacy, which demonstrates both stability and fatigue-resistance.

All compounds demonstrated the desired photopharmacological profile with better activity in their cis configuration at the concentration assayed.

Altogether, these properties encouraged the study of the therapeutic potential of PhotoVDRM in mouse models. The systemic administration of the compound followed by 420 nm irradiation led to rapid reduction in swelling and normalization of skin morphology, unlike dark PhotoVDRM. Remarkably, PhotoVDRM-treated mice did not show hypercalcemia, contrary to conventional VDR agonists, highlighting the impact of localized photoactivation. Finally, molecular analysis corroborated targeted modulation of the immune response consistent with vitamin D’s known effects: pro-inflammatory cytokines were downregulated, while anti-inflammatory markers increased.

In summary, Rovira and colleagues solved a long-standing puzzle of how to use vitamin D’s benefits in psoriasis without inducing undesirable hypercalcemia. They demonstrated the potential of delivering a drug that works only where and when it is needed and can be switched off to avoid any harm. Consequently, this project paves the way toward safer and more precise treatments not only for psoriasis but also beyond, since the concept can be extrapolated to other targets. Photopharmacology, as a field, is maturing from basic proof-of-principle studies into a translational science with real therapeutic implications. The PhotoVDRM story is a clear example of this evolution!
